# Editorial: Optobiomechanics of the eye

**DOI:** 10.3389/fbioe.2025.1727136

**Published:** 2025-11-07

**Authors:** Sabine Kling, Anna Pandolfi, Norberto Lopez‐Gil, Jos Rozema

**Affiliations:** 1 University of Bern, Bern, Switzerland; 2 Politecnico di Milano, Milan, Italy; 3 Universidad de Murcia, Murcia, Spain; 4 Visual Optics Lab Antwerp (VOLANTIS), Faculty of Medicine and Health Sciences, Universiteit Antwerpen, Antwerp, Belgium

**Keywords:** corneal biomechanics, intraocular pressure, lens biomechanics, imaging, modelling

## Introduction

Opto-biomechanics is an interdisciplinary field that focuses on the coupling between optical and mechanical properties in biological systems and on the use of optical approaches to non-invasively study biomechanical behavior. The eye is a complex organ where structural mechanics and optical performance are inherently connected. Understanding this optobiomechanical interplay has become increasingly important in both fundamental research and clinical translation, in particular in the context of corneal cross-linking, refractive surgery, intraocular pressure (IOP) regulation, or lens dynamics.

The eleven contributions featured in this Research Topic span the cornea, sclera, lens, and optic nerve head, presenting new models, novel diagnostic techniques and experimental studies that push forward our ability to diagnose, predict, and potentially treat ocular diseases. This editorial summarizes and synthesizes their insights, highlighting areas of convergence and opportunities for future research.

## Corneal biomechanics

Several studies investigated corneal biomechanics using novel experimental techniques or finite element modeling, particularly in the context of riboflavin/ultraviolet A (UVA) cross-linking (CXL) and laser refractive surgery.

Bell et al. combined synchrotron X-ray scattering, biomechanical testing, and analytical modeling to probe how riboflavin/UVA crosslinking alters the corneal stroma. Interestingly, while cross-linking stiffened the cornea by about 60%, the stiffening was not attributable to changes in individual fibril stiffness. Instead, enhanced fibril interconnectivity and reorientation under load emerged as the dominant mechanism. This better mechanistic understanding demonstrates that therapeutic stiffening relies on mesoscale organization rather than microscale material alterations.

Rix et al. examined Brillouin spectroscopy and polarization sensitive Optical Coherence Tomography (OCT) as a non-invasive measure of corneal biomechanics. By carefully controlling hydration in porcine eyes, the authors demonstrated that Brillouin measurements after CXL are largely confounded by water uptake, in contrast to polarization-sensitive OCT, which more directly captures changes in collagen fiber alignment. Raman spectroscopy further revealed no detectable formation of new molecular cross-links. Together, these results caution against simplistic interpretations of Brillouin data and point toward multimodal approaches for robuster assessments of cross-linking efficacy.

A broader perspective on corneal biomechanics is provided by Pang et al. in a review about finite element (FE) modeling. The authors survey geometrical and constitutive models of the cornea, discuss critical factors influencing numerical outcomes, and highlight validation challenges given the scarcity of human tissue. They underscore FE modeling as an economical and flexible framework for exploring corneal biomechanics and stress the need for more realistic constitutive descriptions.

Refractive surgery and ectasia risk represent another dimension of corneal biomechanics. Zhang et al. trained a random forest model on over 2,600 patients to predict the suitability for Small Incision Lenticule Extraction (SMILE) surgery. With high accuracy (Aread Under the Curve (AUC) = 0.976), the model identified tomographic and biomechanical indices as key discriminators, providing a powerful tool for clinical decision-making. Complementarily, Fantaci et al. used finite-element simulations to investigate whether Photorefractive Keratotomy (PRK), Laser Assisted in-situ Keratomeleusis (LASIK), or SMILE could induce ectasia. Their simulations suggested that surgeries alone are unlikely to *cause* ectasia but may accelerate pre-existing weaknesses, with SMILE having the greatest biomechanical impact on the posterior cornea. Together, these studies highlight how computational models and data-driven tools are valuable for pre-surgical screening and risk assessment.

## Intraocular pressure and biomechanics

The relationship between IOP and ocular biomechanics has been investigated at multiple levels, from tissue-specific alterations to whole-eye deformation responses.

Ma et al. explored how chronic high IOP alters the mechanical properties of the lamina cribrosa (LC) and retinal ganglion cell (RGC) axons. Using atomic force microscopy on a rat glaucoma model, the authors documented a time-dependent reduction in stiffness of both LC glial tissue and RGC axons, with up to 80% loss in modulus over 12 weeks. This weakening likely contributes to axonal vulnerability and progressive glaucomatous damage, emphasizing the need for therapies that preserve or restore LC integrity.

Extending the focus from local tissue mechanics to global ocular responses, two other studies applied air-puff tonometry to connect corneal and scleral responses to IOP. Redaelli et al. developed a computational model to simulate corneal deformation under air-puff tonometry, addressing the fact that traditional measures are confounded by corneal thickness and the tissue’s mechanical properties. By shifting the analysis toward the timing of maximum apex velocity, the authors propose a more reliable IOP estimator less dependent on corneal properties. Using the same air-puff tonometry, De La Hoz et al. examined scleral biomechanics in rabbits in combination with computational modeling. Their findings showed scleral stiffness strongly influences deformation responses, suggesting that this non-invasive tool could provide valuable biomechanical markers for myopia and glaucoma risk evaluation.

## Lens biomechanics

Although crucial to the process of accommodation, the biomechanical behavior of the crystalline lens is not yet entirely understood. Dahaghin et al. introduced a biomechanical model of lens “wobbling,” the oscillatory motion that occurs after rapid eye movement. By combining Purkinje image analysis with optobiomechanical simulations, the authors reproduced oscillation frequencies and damping factors observed *in vivo*, while also revealing subject-specific variability. This work sets the stage for personalized lens models that could deepen our understanding of ocular biomechanical mechanisms.

Tahsini et al. examined how preservation methods alter the mechanical properties of *ex vivo* porcine lenses. Using optical coherence elastography and inverse finite-element analysis, the authors showed that freezing significantly alters cortical and nuclear strains, while refrigeration preserves the lens’ mechanical properites best. Removal of the capsule changed the strain distribution across lens nucleus and cortex. These findings are essential for interpreting *ex vivo* experiments and designing standardized protocols for lens biomechanics research.

## Conclusion

Taken together, these eleven contributions provide a rich insight in the evolving field of optobiomechanics.

From synchrotron scattering to, air-puff tonometry, OCT elastography, machine learning and numerical simulations, diverse methods are converging to give a more complete picture of ocular biomechanics. Studies on corneal cross-linking, scleral stiffness, and lamina cribrosa degradation deepen our understanding of disease and treatment mechanisms. Computational models and predictive algorithms are directly informing refractive surgery screening and IOP measurement, while non-invasive imaging modalities offer prospects for routine biomechanical monitoring.

The continued growth of optobiomechanics will rely on interdisciplinary collaborations across physics, biology, engineering, and clinical sciences, ultimately driving innovations that improve patient care and visual performance.

